# Prevalence and determinants of asymmetric septal hypertrophy (ASH) in diseases of left ventricular hypertrophy (LVH)

**DOI:** 10.1093/ehjci/jeag203

**Published:** 2026-08-03

**Authors:** Hunain Shiwani, João Augusto, Constantin-Cristian Topriceanu, Adam Ioannou, Stefania Rosmini, Viviana Maestrini, Rebecca Hughes, Kristopher Knott, Anish Bhuva, Benjamin Dowsing, Jessica Artico, Iain Pierce, Thomas Treibel, James Howard, Charlotte Manisty, Peter Kellman, Rebecca Kozor, Richard Steeds, Gabriella Captur, Saidi Mohiddin, Luis Lopes, Marianna Fontana, James Moon, Rhodri Davies

**Affiliations:** Institute of Cardiovascular Science, University College London, 62 Huntley St, London WC1E 6DD, UK; Barts Heart Centre, The Cardiovascular Magnetic Resonance Imaging Unit and the Inherited Cardiovascular Diseases Unit, St Bartholomew’s Hospital, West Smithfield, London EC1A 7BE, UK; Institute of Cardiovascular Science, University College London, 62 Huntley St, London WC1E 6DD, UK; Barts Heart Centre, The Cardiovascular Magnetic Resonance Imaging Unit and the Inherited Cardiovascular Diseases Unit, St Bartholomew’s Hospital, West Smithfield, London EC1A 7BE, UK; Institute of Cardiovascular Science, University College London, 62 Huntley St, London WC1E 6DD, UK; Institute of Cardiovascular Science, University College London, 62 Huntley St, London WC1E 6DD, UK; National Amyloidosis Centre, Division of Medicine, University College London, Royal Free Hospital, Rowland Hill Street, London NW3 2PF, UK; Barts Heart Centre, The Cardiovascular Magnetic Resonance Imaging Unit and the Inherited Cardiovascular Diseases Unit, St Bartholomew’s Hospital, West Smithfield, London EC1A 7BE, UK; Department of Cardiology, King’s College Hospital NHS Foundation Trust, Denmark Hill, London SE5 9RS, UK; Department of Clinical, Internal Medicine, Anesthesiological and Cardiovascular Sciences, Sapienza University of Rome, Rome, Italy; Institute of Cardiovascular Science, University College London, 62 Huntley St, London WC1E 6DD, UK; Barts Heart Centre, The Cardiovascular Magnetic Resonance Imaging Unit and the Inherited Cardiovascular Diseases Unit, St Bartholomew’s Hospital, West Smithfield, London EC1A 7BE, UK; Institute of Cardiovascular Science, University College London, 62 Huntley St, London WC1E 6DD, UK; Barts Heart Centre, The Cardiovascular Magnetic Resonance Imaging Unit and the Inherited Cardiovascular Diseases Unit, St Bartholomew’s Hospital, West Smithfield, London EC1A 7BE, UK; Institute of Cardiovascular Science, University College London, 62 Huntley St, London WC1E 6DD, UK; Barts Heart Centre, The Cardiovascular Magnetic Resonance Imaging Unit and the Inherited Cardiovascular Diseases Unit, St Bartholomew’s Hospital, West Smithfield, London EC1A 7BE, UK; Institute of Cardiovascular Science, University College London, 62 Huntley St, London WC1E 6DD, UK; Barts Heart Centre, The Cardiovascular Magnetic Resonance Imaging Unit and the Inherited Cardiovascular Diseases Unit, St Bartholomew’s Hospital, West Smithfield, London EC1A 7BE, UK; Institute of Cardiovascular Science, University College London, 62 Huntley St, London WC1E 6DD, UK; Barts Heart Centre, The Cardiovascular Magnetic Resonance Imaging Unit and the Inherited Cardiovascular Diseases Unit, St Bartholomew’s Hospital, West Smithfield, London EC1A 7BE, UK; Institute of Cardiovascular Science, University College London, 62 Huntley St, London WC1E 6DD, UK; Barts Heart Centre, The Cardiovascular Magnetic Resonance Imaging Unit and the Inherited Cardiovascular Diseases Unit, St Bartholomew’s Hospital, West Smithfield, London EC1A 7BE, UK; Institute of Cardiovascular Science, University College London, 62 Huntley St, London WC1E 6DD, UK; Barts Heart Centre, The Cardiovascular Magnetic Resonance Imaging Unit and the Inherited Cardiovascular Diseases Unit, St Bartholomew’s Hospital, West Smithfield, London EC1A 7BE, UK; National Heart and Lung Institute, Imperial College London, London SW7 2AZ, UK; Institute of Cardiovascular Science, University College London, 62 Huntley St, London WC1E 6DD, UK; Barts Heart Centre, The Cardiovascular Magnetic Resonance Imaging Unit and the Inherited Cardiovascular Diseases Unit, St Bartholomew’s Hospital, West Smithfield, London EC1A 7BE, UK; National Heart, Lung, and Blood Institute, National Institutes of Health Bethesda, USA; Kolling Institute, Royal North Shore Hospital, and University of Sydney, Sydney, Australia; Department of Cardiology, Queen Elizabeth Hospital Birmingham, University Hospitals Birmingham NHS Foundation Trust and University of Birmingham, Birmingham, UK; Institute of Cardiovascular Science, University College London, 62 Huntley St, London WC1E 6DD, UK; MRC Unit of Lifelong Health and Ageing, University College London, 1-19 Torrington Place, Fitzrovia, London WC1E 7HB, UK; Inherited Heart Muscle Conditions Clinic, Department of Cardiology, Royal Free London NHS Foundation Trust, Pond Street, Hampstead, London NW3 2QG, UK; Barts Heart Centre, The Cardiovascular Magnetic Resonance Imaging Unit and the Inherited Cardiovascular Diseases Unit, St Bartholomew’s Hospital, West Smithfield, London EC1A 7BE, UK; Institute of Cardiovascular Science, University College London, 62 Huntley St, London WC1E 6DD, UK; Barts Heart Centre, The Cardiovascular Magnetic Resonance Imaging Unit and the Inherited Cardiovascular Diseases Unit, St Bartholomew’s Hospital, West Smithfield, London EC1A 7BE, UK; Institute of Cardiovascular Science, University College London, 62 Huntley St, London WC1E 6DD, UK; National Amyloidosis Centre, Division of Medicine, University College London, Royal Free Hospital, Rowland Hill Street, London NW3 2PF, UK; Institute of Cardiovascular Science, University College London, 62 Huntley St, London WC1E 6DD, UK; Barts Heart Centre, The Cardiovascular Magnetic Resonance Imaging Unit and the Inherited Cardiovascular Diseases Unit, St Bartholomew’s Hospital, West Smithfield, London EC1A 7BE, UK; Institute of Cardiovascular Science, University College London, 62 Huntley St, London WC1E 6DD, UK; Barts Heart Centre, The Cardiovascular Magnetic Resonance Imaging Unit and the Inherited Cardiovascular Diseases Unit, St Bartholomew’s Hospital, West Smithfield, London EC1A 7BE, UK

**Keywords:** hypertrophic cardiomyopathy, asymmetric septal hypertrophy, left ventricular hypertrophy, cardiovascular magnetic resonance, amyloidosis, aortic stenosis

## Abstract

**Aims:**

To determine the prevalence of asymmetric septal hypertrophy (ASH) across health and in diseases associated with left ventricular hypertrophy (LVH), including hypertrophic cardiomyopathy (HCM).

**Methods and results:**

We analysed two complementary datasets. First, a large UK Biobank healthy reference cohort (*n* = 4020) was used to model demographic determinants of septal-to-lateral wall thickness ratio (SLR) and to provide the reference group for age-, sex-, and body surface area-adjusted comparisons. Second, a multi-cohort clinical CMR dataset included 1655 subjects comprising local healthy volunteers, athletes, patients with hypertension, aortic stenosis, Fabry disease, amyloid light chain (AL) amyloidosis, amyloid transthyretin (ATTR) amyloidosis, and HCM. LVH was defined by maximum wall thickness (MWT) ≥15 mm and asymmetry defined as SLR≥1.3. Left ventricular morphology was classified as normal, asymmetric remodelling (SLR≥1.3; MWT<15 mm), symmetric hypertrophy (SLR<1.3; MWT≥15 mm) or ASH (SLR≥1.3; MWT≥15 mm). ASH was highly prevalent in HCM (61%) but was also observed in ATTR amyloidosis (37%), AS (26%), and AL amyloidosis (23%). In the healthy reference cohort, a higher septal-to-lateral ratio was independently associated with older age, female sex, and larger body surface area (*P* < 0.001). After adjustment for these demographic factors, only HCM retained a clinically meaningful excess in septal asymmetry (ΔSLR = +0.27; *P* < 0.001). In contrast, the apparent asymmetry in non-HCM cohorts was partly explained by demographic variation. After adjustment, only HCM showed a clinically meaningful excess in SLR, whereas other disease cohorts showed either no material difference or small negative differences relative to healthy controls.

**Conclusion:**

ASH is a common but non-specific finding in diseases associated with LVH. Septal asymmetry increases with age and body-size and is more pronounced in women. After adjusting for age, sex, and body-size, disproportionate septal asymmetry was most characteristic of HCM.

## Graphical Abstract

**Graphical Abstract:**
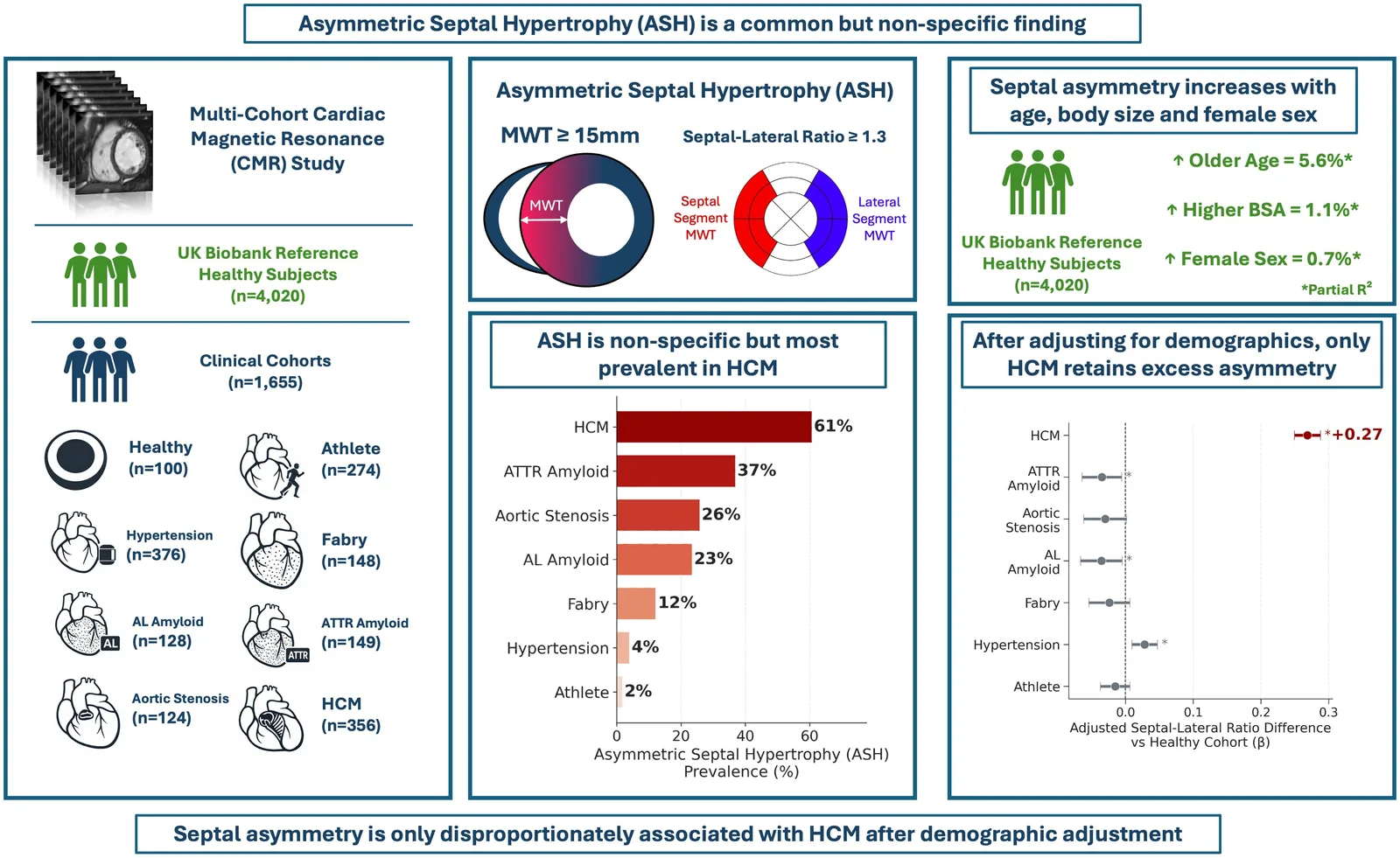
Asymmetric septal hypertrophy (ASH) is common but non-specific across diseases of left ventricular hypertrophy. Although ASH is most prevalent in hypertrophic cardiomyopathy (HCM), septal asymmetry also increases with older age, larger body size, and female sex. After adjustment for these demographic factors, only HCM retains a clinically meaningful excess in septal asymmetry.

**See the editorial comment for this article ‘Not all asymmetry is equal: asymmetric septal hypertrophy across hypertrophic cardiomyopathy, amyloidosis and beyond’, by N. Ciocca and C. Gräni, https://doi.org/10.1093/ehjci/jeag214.**

## Introduction

Left ventricular hypertrophy (LVH) can be an adaptive response to chronic haemodynamic stress, stemming from either physiological (such as exercise) or pathological stressors (increased afterload due to systemic hypertension or aortic stenosis (AS)).^1^ LVH also manifests in primary heart muscle diseases (i.e. cardiomyopathies) which encompass hypertrophic cardiomyopathy (HCM), infiltrative heart disease, and storage and metabolic disorders.^2^ The pattern of hypertrophy can contribute and provide important diagnostic clues to the underlying aetiology.^3^ Asymmetric septal hypertrophy (ASH) is classically associated with HCM, but septal asymmetry is not specific to HCM and has also been documented in other diseases such as transthyretin cardiac amyloidosis (ATTR).^4,5^

ASH was first described in the 1950s as a feature of HCM.^6,7^ While the exact mechanisms behind the development of asymmetrical hypertrophy are not fully understood, it is thought to be related to imbalances in cardiac workload and muscle growth signalling pathways.^8,9^ An increase in absolute maximum wall thickness (MWT) is associated with a higher risk of sudden cardiac death^10–12^ and is thought to be a contributing factor to left ventricular outflow tract obstruction (LVOTO).^13^ While the most common region of hypertrophy is the basal septal wall, other morphological phenotypes have been identified including concentric hypertrophy, apical HCM, mid-cavitary HCM, mass-like HCM, and non-contiguous HCM.^14^ However, HCM phenocopies also exhibit ASH.

The extent of asymmetry can be quantified using the septal–lateral ratio (SLR): the ratio of maximum septal wall thickness (anteroseptal or inferoseptal) to maximum lateral wall thickness (anterolateral or inferolateral). Early work based on autopsy studies, suggested a SLR threshold of 1.5.^7^ With the advent of non-invasive imaging, an SLR of 1.3 was proposed as a pragmatic threshold to describe septal asymmetry on echocardiography, although this should be regarded as context-dependent rather than a definitive disease boundary.^15^ However, these thresholds are derived from relatively small cohorts and may not generalize across the spectrum of diseases associated with LVH. Furthermore, conventional assessment relies on manual measurements, which are subject to inter-observer variability and may limit reproducibility.

As a result, the prevalence and distribution of LVH patterns across diseases have not been systematically quantified at scale. We aimed to characterize the prevalence and asymmetry of LVH in a large set of subjects across multiple diseases. To achieve this, we applied a previously validated automated method for wall thickness quantification that has demonstrated high precision and scalability.^16,17^ We analysed cardiac magnetic resonance (CMR) cine images from multiple centres, including cohorts with HCM, Fabry disease, cardiac light chain amyloidosis (AL), and cardiac transthyretin amyloidosis (ATTR). For comparison, we included healthy volunteers, athletes (physiological adaptation) and arterial hypertension and severe AS (as models of increased afterload).

## Methods

All data were acquired from participants previously recruited into research studies with written informed consent and National Health Service Research Ethics Committee approval, in accordance with the Declaration of Helsinki.

### Data

This study used two complementary CMR datasets with distinct roles.

The first dataset was a large UK Biobank healthy reference cohort. This was used to model the relationship between SLR and demographic variables in health, specifically age, sex, and body surface area (BSA), and to provide the healthy reference group for adjusted comparisons across cohorts. The UK Biobank cohort is substantially larger and better suited to estimating demographic determinants of septal asymmetry.

The second dataset was a multi-cohort clinical dataset comprising patients with diseases associated with LVH, athletes, and local healthy volunteers. This dataset was used to describe the prevalence of morphology categories across clinically relevant comparator groups. The local healthy volunteer cohort provided a complementary healthy comparator with a younger mean age and broader demographic variation than the UK Biobank reference cohort, allowing the findings to be assessed across a wider spectrum of age and body size. Thus, whereas UK Biobank served as the primary healthy reference population, the local healthy volunteers provided a smaller local comparator cohort within the clinical dataset.

All subjects underwent CMR performed using standard protocols^18^ and all cine images were acquired using balanced steady-state free precession sequences. Unless otherwise stated, all patients were scanned using a Siemens 1.5T scanner (Avanto or Aera; Siemens Healthcare, Erlangen, Germany). Typical slice thickness was 8 mm and typical pixel spacing was 1.4 × 1.4 mm.

### Healthy UK Biobank subjects

To investigate demographic determinants of septal asymmetry, we used a healthy subset of subjects recruited to the UK Biobank, enrolled in the imaging sub-study who underwent CMR scanning were taking no medications and were free from hypertension, diabetes, hypercholesterolaemia, peripheral vascular disease, coronary artery disease, and cerebrovascular disease.

### Local healthy volunteers

A separate smaller healthy volunteer comparator cohort was used. Local healthy volunteers were recruited across St Bartholomew’s Hospital, Royal Free Hospital, and the London Heart Hospital. All volunteers had no history of cardiovascular disease, took no medications, and had a normal ECG.

### Athletes

Athletes were recruited from St Bartholomew’s Hospital, Royal Free Hospital, London Heart Hospital and St George’s Hospital. Athletes were aged >40 years and had undertaken long-term endurance exercise, defined as running ≥10 miles per week or cycling ≥30 miles per week for ≥10 years, with participation in ≥10 endurance events over a 10-year period. Individuals with known cardiomyopathy or cardiovascular disease were not included in the athlete cohort.

### Hypertensive patients

Hypertensive patients were a subset of subjects recruited to the UK Biobank, enrolled in the imaging sub-study who underwent CMR scanning while taking ≥3 antihypertensive medications. To remove complicating factors, patients were only selected if they were free from diabetes, hypercholesterolaemia, peripheral vascular disease, coronary artery disease, and cerebrovascular disease.

### Aortic stenosis

AS patients with severe AS (aortic valve velocity of ≥4 m/s; mean gradient of 40 mm Hg or greater; or aortic valve area less than 1.0 cm^2^) were recruited from St Bartholomew’s Hospital, Royal Free Hospital, and the London Heart Hospital.

### Fabry disease

Fabry disease patients were recruited from the Lysosomal Storage Disorders Unit at the Royal Free Hospital, London as part of the prospective Fabry400 study. Consecutive Fabry disease patients were included if they were aged ≥18 years, had a confirmed GLA mutation, and underwent multiparametric CMR with same-day ECG. Patients aged <18 years, those with standard contraindications to CMR, and patients with known pregnancy were excluded.^19^

### Cardiac amyloidosis

AL and wild-type ATTR amyloidosis were recruited from the UK National Amyloidosis Centre. A diagnosis of TTR amyloid was made based on clinical features, personal, and family history, CMR +/− genetic findings, along with grade 2 or 3 cardiac uptake on 99mTc-DPD scintigraphy, in the absence of monoclonal gammopathy. In cases where monoclonal gammopathy was present, a positive cardiac biopsy for TTR was also required.^4^ For cardiac AL amyloidosis, the definition included the presence of typical features on CMR and a biopsy-proven systemic AL amyloidosis on either cardiac or non-cardiac biopsy. All patients with cardiac amyloidosis also exhibited typical features on LGE imaging.

### Hypertrophic cardiomyopathy

Patients with HCM were recruited from the Inherited Cardiovascular Conditions service at St Bartholomew’s Hospital, where they were assessed, diagnosed, and treated by specialist inherited cardiac conditions cardiologists. HCM was diagnosed as otherwise unexplained LV hypertrophy, integrating family history, ECG, cardiac imaging, loading conditions, and genetic testing where available. Alternative causes of LV hypertrophy were excluded through routine clinical assessment.

### Wall thickness measurement

The approach to wall thickness measurement is illustrated in *Figure 1*. The endocardial and epicardial borders of the left ventricle were delineated on standard short axis cine stacks using a convolutional neural network.^16^ End-diastole was chosen as the cardiac phase with largest LV blood pool volume and wall thickness was measured between the two contours as previously described.^17^ Papillary muscles were excluded from myocardial wall-thickness measurement according to the automated contouring approach. SLR was calculated from the MWT in the basal and mid septal segments divided by the MWT in the basal and mid lateral segments. Basal slices were included only where a circumferential LV myocardial contour was present; slices dominated by the LV outflow tract or mitral valve plane were not used for segmental wall-thickness calculation. SLR was measured as the ratio between the MWT in the basal and mid septal (from either anteroseptal or inferoseptal) segments and the basal and mid lateral (anterolateral and inferolateral) segments.

**Figure 1:**
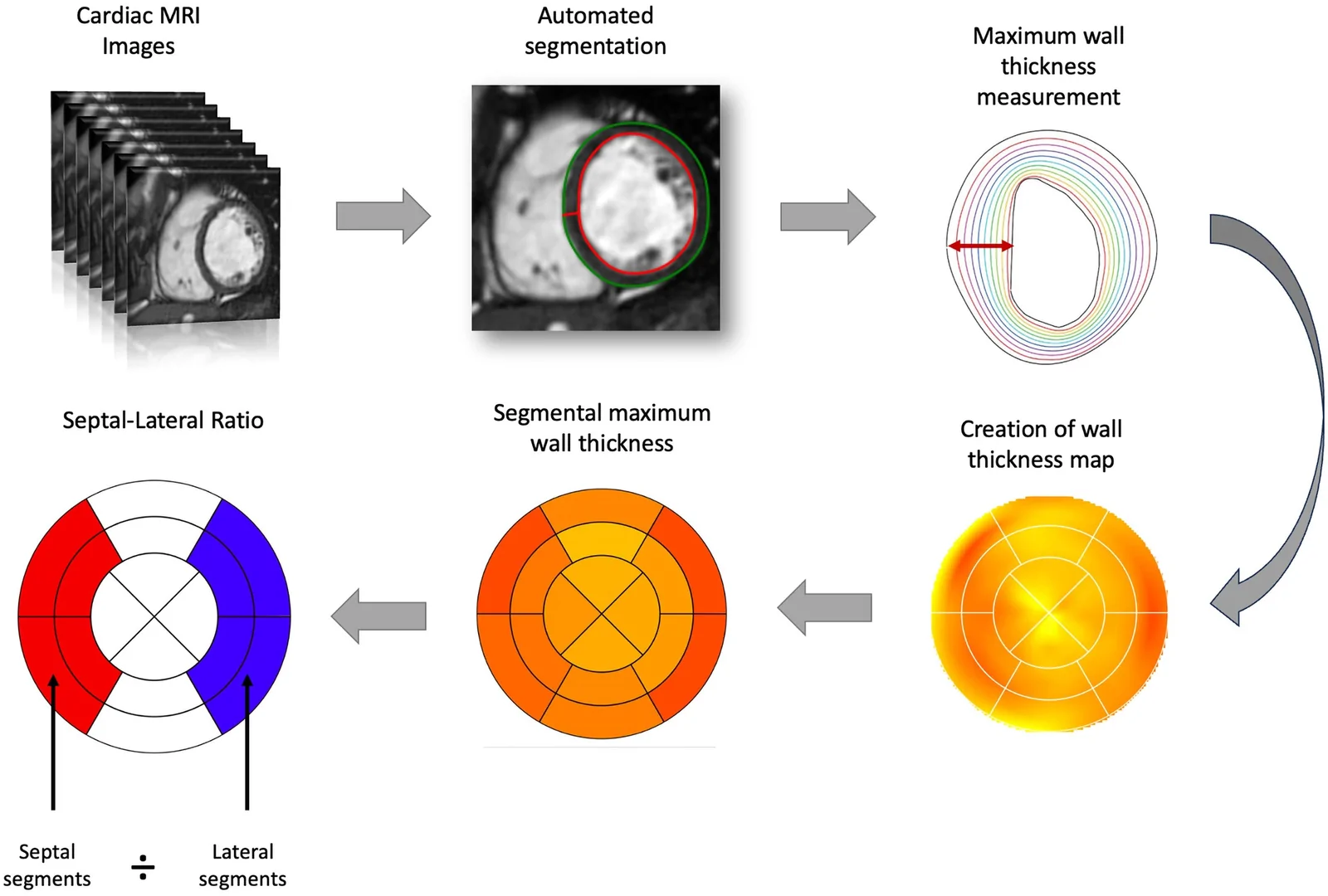
Overview figure of wall thickness measurements. Short axis (SAX) cine stack in diastole, automated segmentation to produce epicardial and endocardial contours, calculating maximum wall thickness (MWT) measurements using contours, creation of wall thickness map from MWT measurements, 16-segment American Heart Association (AHA) map with a defined maximum wall thickness for each segment and four septal segments highlighted in red and four lateral segments highlighted in blue. The septal–lateral ratio is derived by identifying the thickest MWT of each segment group and dividing the septal MWT by the lateral MWT.

### Statistical analysis

Continuous variables were reported as mean ± standard deviation, while categorical variables were presented as counts and percentages. The demographic and clinical characteristics of the study cohorts were compared using one-way analysis of variance (ANOVA) for continuous variables and χ^2^ tests for categorical variables. When the overall ANOVA indicated differences, we performed *post hoc* pairwise comparisons of continuous variables (age, BSA, MWT, and SLR) using Tukey’s honestly significant difference test.

A descriptive categorical analysis was used to summarize left ventricular morphology across cohorts. LVH was defined as MWT ≥ 15 mm and asymmetry as SLR ≥ 1.3. Participants were classified into four categories: normal (no LVH, symmetric), asymmetric remodelling (no LVH, asymmetric), symmetric hypertrophy (LVH, symmetric), and ASH (LVH, asymmetric). These are illustrated in *Figure 2*. The prevalence of these categories was summarized by cohort, and proportions were compared using χ^2^ tests. Although we have used a threshold of ≥1.3, as there are varying thresholds in the literature, supplementary analysis was performed using a threshold of ≥1.5.

**Figure 2:**
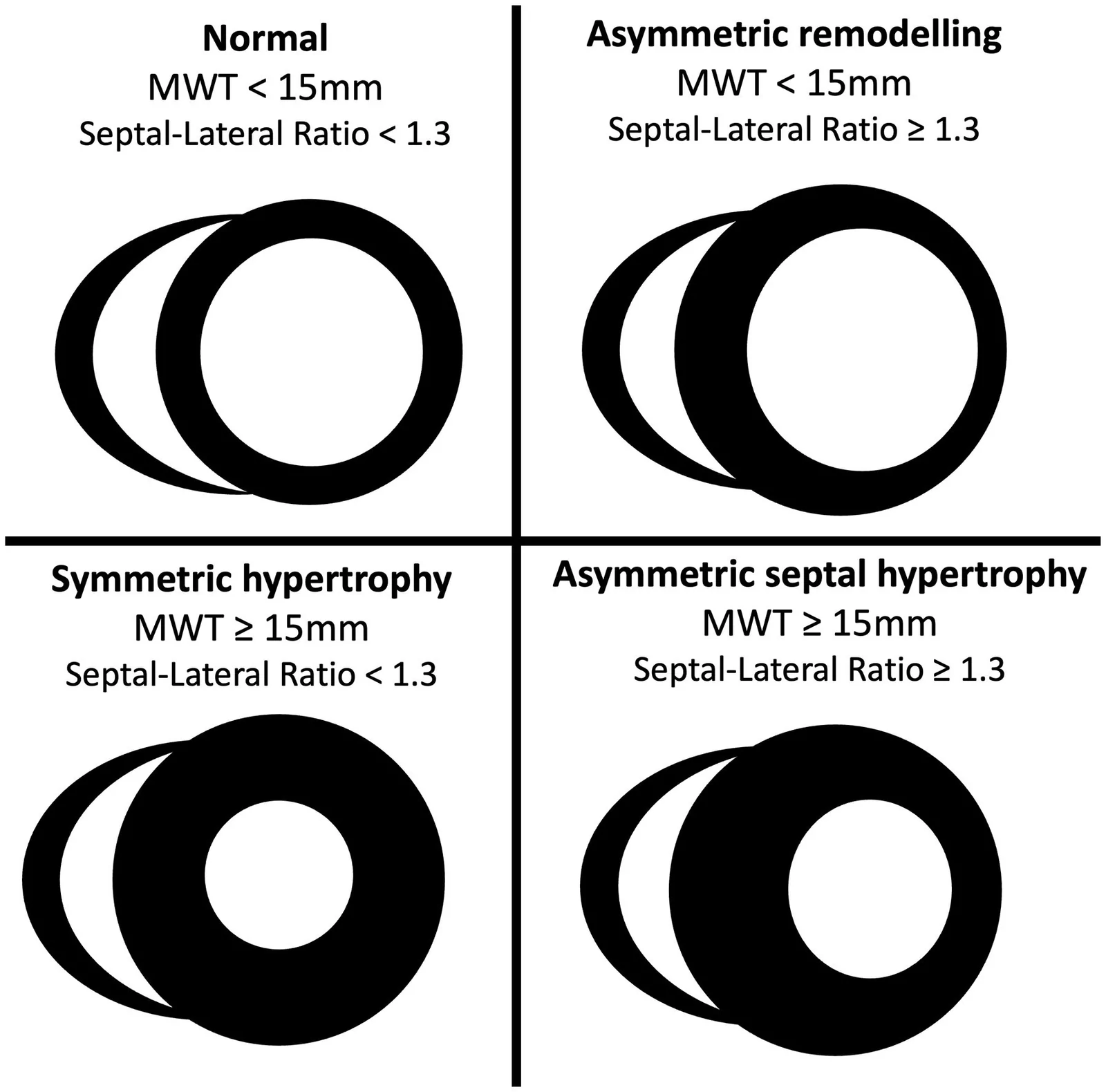
LVH was defined as maximum wall thickness ≥15 mm, and asymmetry was defined as septal–lateral ratio ≥1.3. Patients were grouped into one of four categories: normal (no LVH, symmetric), asymmetric remodelling (no LVH, asymmetric), symmetric hypertrophy (LVH, symmetric), and asymmetric septal hypertrophy (ASH) (LVH, asymmetric).

An adjusted continuous analysis was performed using multivariable linear regression with SLR as a continuous dependent variable. In the healthy UK Biobank cohort, SLR was regressed on age, BSA, and sex to identify independent determinants of septal asymmetry. Standardized regression coefficients (*β*) and partial *R*^2^ values are reported.

To compare disease cohorts with healthy reference subjects, SLR was modelled in a pooled dataset including all cohorts using multivariable linear regression with age, BSA, sex, and cohort as predictors. UK Biobank healthy volunteers were used as the reference group. Regression coefficients for cohort terms represent the adjusted difference in mean SLR relative to the healthy reference cohort (ΔSLR), with 95% confidence intervals and *P* values reported.

As a sensitivity analysis, interactions between demographic predictors were assessed in the UK Biobank healthy reference cohort by adding age × sex, age × BSA and sex × BSA interaction terms to the additive model. Model fit was compared with the primary additive model using change in *R*^2^, likelihood-ratio testing and Akaike information criterion (AIC).

Model assumptions for linear regression were assessed by inspection of residual-vs.-fitted plots and Q–Q plots. As a sensitivity analysis, restricted cubic spline models were explored for age and BSA to assess possible non-linearity. The linear model was retained as the primary analysis for interpretability. All analyses were performed in Python 3.8 (Python Software Foundation, Oregon, USA) using SciPy and statsmodels libraries.

## Results

### Demographics

The UK Biobank healthy reference cohort group comprised 4020 participants [mean age 61 ± 7 years; 50% men (*n* = 1998)]. Mean BSA was 1.8 ± 0.2 m^2^. CMR measures were: end-diastolic volume (EDV) 142 ± 32 mL, end-systolic volume (ESV) 45 ± 16 mL, left ventricular mass (LVM) 93 ± 24 g, and MWT 10.7 ± 1.9 mm. The septal–lateral wall thickness ratio averaged 1.3 ± 0.2 (septal MWT 10.6 ± 1.9 mm, lateral MWT 8.5 ± 1.4 mm). This cohort served as the healthy reference for all adjusted analyses of SLR across disease groups.

In total, 1655 subjects were recruited across disease and validation cohorts: local healthy volunteers (*n* = 100), athletes (*n* = 274), patients with hypertension (*n* = 376), AS (*n* = 124), Fabry disease (*n* = 148), AL amyloidosis (*n* = 128), ATTR amyloidosis (*n* = 149), and HCM (*n* = 356). Mean age ranged from 44 ± 13 years in the Fabry disease cohort to 73 ± 10 years in the ATTR amyloidosis cohort. The proportion of male participants ranged from 39% in the Fabry disease cohort to 81% in the ATTR amyloidosis cohort. Mean BSA ranged from 1.79 ± 0.19 m^2^ in athletes to 1.99 ± 0.24 m^2^ in the HCM cohort.

Standard cardiac measures for each cohort are presented in *Table 1*. EDV was highest in athletes (171 ± 38 mL) and lowest in local healthy volunteers (146 ± 33 mL). Left ventricular mass was greatest in patients with ATTR amyloidosis (189 ± 66 g) and lowest in local healthy volunteers (90 ± 23 g). Mean MWT was highest in patients with HCM (19.5 ± 5.0 mm) and lowest in local healthy volunteers (9.9 ± 1.8 mm). The mean septal-to-lateral wall-thickness ratio ranged from 1.13 ± 0.15 in local healthy volunteers to 1.50 ± 0.34 in patients with HCM.

**Table 1:** Standard measures of cardiac structure and function of all cohort

	UK Biobank healthy volunteer (*n* = 4020)	Healthy volunteer (*n* = 100)	Athlete (*n* = 274)	Hypertension (*n* = 376)	Aortic stenosis (*n* = 124)	Fabry (*n* = 148)	AL amyloid (*n* = 128)	ATTR amyloid (*n* = 149)	Hypertrophic cardiomyopathy (*n* = 356)
**Age (years)**	61 (7)	48 (14)	54 (7)	65 (6)	71 (10)	44 (13)	65 (10)	73 (10)	56 (14)
**Sex (*n*) (Males %)**	1998 (50%)	50 (50%)	123 (45%)	228 (61%)	86 (69%)	58 (39%)	81 (63%)	121 (81%)	268 (75%)
**BSA (m^2^)**	1.8 (0.2)	1.87 (0.23)	1.79 (0.19)	1.98 (0.21)	1.93 (0.21)	1.83 (0.21)	1.85 (0.23)	1.90 (0.22)	1.99 (0.24)
**EDV (mLs)**	142 (32)	146 (33)	171 (38)	150 (34)	154 (51)	156 (35)	144 (38)	153 (41)	154 (42)
**ESV (mls)**	45 (16)	50 (15)	57 (17)	44 (16)	56 (43)	44 (16)	55 (28)	69 (35)	45 (26)
**LVM (g)**	93 (24)	90 (23)	110 (29)	105 (28)	155 (44)	138 (69)	164 (61)	189 (66)	174 (72)
**MWT (mm)**	10.7 (1.9)	9.9 (1.8)	11.0 (1.8)	12.2 (2.2)	16.9 (5.1)	13.6 (5.0)	17.1 (3.7)	19.8 (4.8)	19.5 (5.0)
**Septal–lateral ratio**	1.3 (0.2)	1.13 (0.15)	1.20 (0.14)	1.30 (0.16)	1.26 (0.18)	1.15 (0.19)	1.22 (0.20)	1.26 (0.19)	1.50 (0.34)
**Septal MWT (mm)**	10.6 (1.9)	9.8 (1.9)	10.9 (1.8)	12.2 (2.1)	16.1 (3.9)	13.2 (4.8)	16.8 (3.8)	19.6 (4.8)	19.2 (5.0)
**Lateral MWT (mm)**	8.5 (1.4)	8.7 (1.4)	9.2 (1.4)	9.4 (1.7)	12.9 (2.9)	11.4 (3.3)	14.0 (3.3)	15.8 (4.2)	13.3 (4.0)

AL, amyloid light chain; ATTR, amyloid transthyretin; HCM, hypertrophic cardiomyopathy.

### Determinants of asymmetry in healthy Controls

In UK Biobank healthy participants, higher SLR was independently associated with older age, larger body size, and female sex in a multivariable linear model. Standardized effect sizes (*β*) and unique variance explained (partial *R*^2^) were: age *β* = +0.24; partial *R*^2^ = 5.6%, BSA *β* = +0.14; partial *R*^2^ = 1.1%, and female sex β = +0.11; partial *R*^2^ = 0.7% (all *P* < 0.001). The model explained *R*^2^ = 6% of SLR variance. These are shown in *Figure 3*.

**Figure 3:**
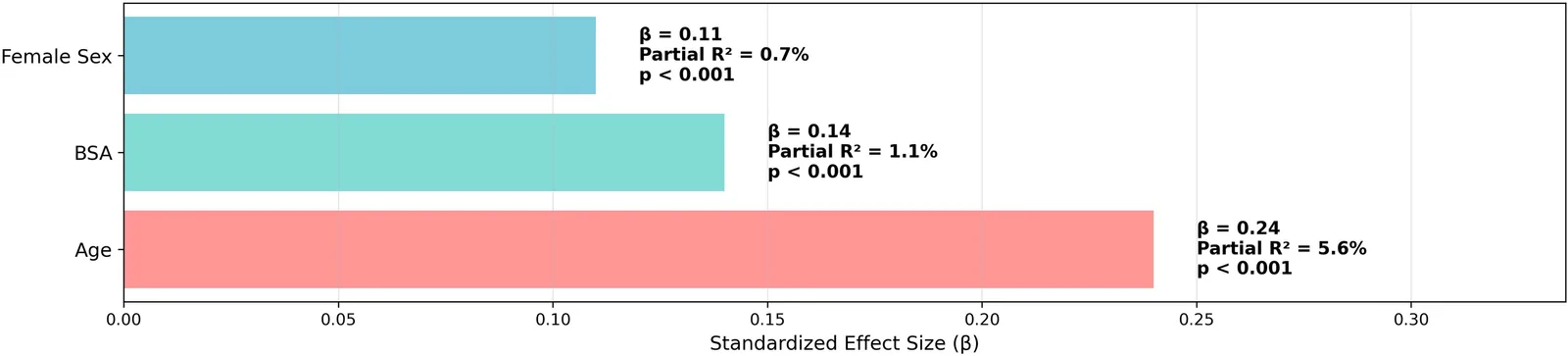
Determinants of septal asymmetry in healthy adults. Standardized β coefficients from OLS regression of the septal-to-lateral wall thickness ratio (SLR) on age, body surface area (BSA), and sex in healthy UK Biobank participants (*n* = 4020). All predictors were positively associated with higher SLR-age strongest (*β*=0.24; partial *R*^2^ = 5.6%), then BSA (*β*=0.14; 1.1%), and female sex (*β*=0.11; 0.7%); all *P* < 0.001.

The UK Biobank participants had a higher mean SLR than the local healthy volunteer cohort. This difference was partly attributable to demographic structure, particularly age. In UK Biobank healthy participants, age was the strongest independent determinant of SLR (*b* = 0.0051 per year, standardized *β*=0.240, partial *R*^2^ = 5.58%, *P* < 0.001), with smaller independent contributions from BSA and sex. However, demographic factors did not fully explain the difference between healthy cohorts: in a combined healthy-subject model adjusting for age, sex, and BSA, local healthy volunteers retained a lower adjusted SLR than UK Biobank healthy participants (adjusted difference −0.0466, 95% CI −0.0777 to −0.0154, *P* = 0.003).

We also assessed whether demographic predictors interacted with one another in the UK Biobank healthy reference cohort. Adding age × sex, age × BSA and sex × BSA interaction terms significantly improved model fit compared with the additive model, but the incremental explanatory value was small (ΔR^2^ = 0.0044; likelihood-ratio χ^2^ = 18.87, df = 3; *P* < 0.001; ΔAIC = −12.87).

### Baseline LV morphology

Threshold-defined ASH was most prevalent in HCM (61%), followed by ATTR amyloidosis (37%), AS (26%), AL amyloidosis (23%), Fabry disease (12%), hypertension (4%), athletes (2%), and local healthy volunteers (0%). Asymmetric remodelling without LVH was most frequent in the hypertension cohort (42%) and least frequent in the Fabry disease cohort (6%). Symmetric hypertrophy was most common in AL amyloidosis (49%) and least common in athletes (1%) and local healthy volunteers (1%). These findings are shown in *Figure 4*.

**Figure 4:**
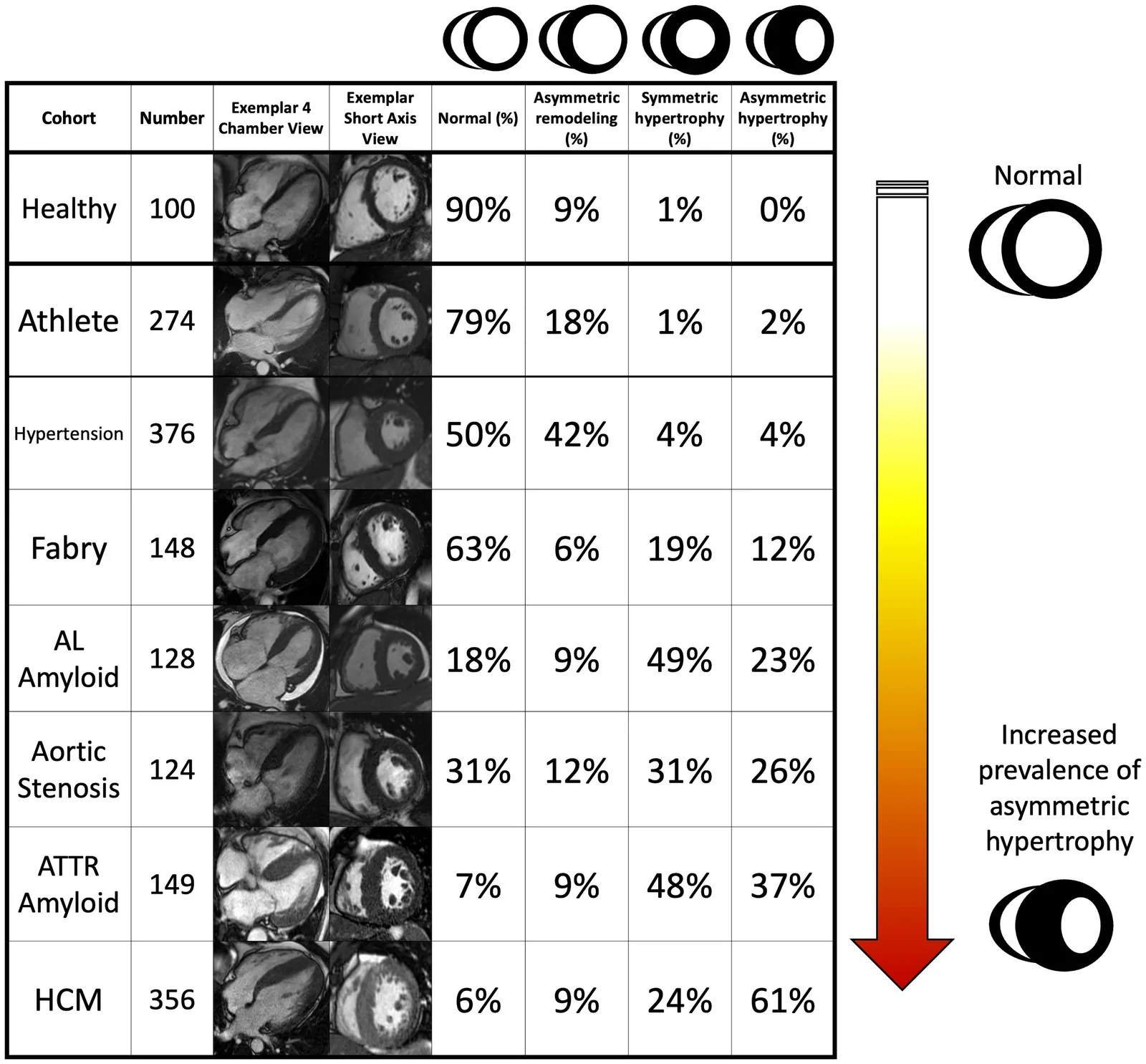
Distribution of hypertrophy patterns in various cohorts, including healthy volunteers, athlete, hypertension, Fabry disease, AL amyloid, aortic stenosis, ATTR amyloid, and HCM patients. The percentage of patients in each cohort with normal cardiac structure, asymmetric remodelling, symmetric hypertrophy, and asymmetric septal hypertrophy (ASH) is shown. The figure also includes exemplar CMR 4-chamber and short-axis views of patients closest to the median septal–lateral ratio value within the cohort. AL, amyloid light chain; ATTR, amyloid transthyretin; HCM, hypertrophic cardiomyopathy).

### Adjusted differences in septal asymmetry across cohorts

After adjustment for BSA, age, and sex, HCM showed a substantially higher SLR (ΔSLR = +0.27; 95% CI +0.23 to +0.31; *P* < 0.001) compared with the UK Biobank healthy control population. A small positive difference was seen in hypertension (ΔSLR = +0.03; 95% CI +0.01 to +0.05; *P* = 0.002). Small negative differences were observed in AL amyloidosis (ΔSLR = −0.04; 95% CI −0.07 to −0.00; *P* = 0.044) and ATTR amyloidosis (ΔSLR = −0.04; 95% CI −0.07 to −0.00; *P* = 0.035). No material difference was seen in AS (ΔSLR = −0.03; 95% CI −0.06 to +0.00; *P* = 0.066), athletes (ΔSLR = −0.02; 95% CI −0.03 to +0.00; *P* = 0.092), and Fabry disease (ΔSLR = −0.02; 95% CI −0.05 to +0.01; *P* = 0.138). The local healthy volunteer cohort had a lower adjusted SLR than the UK Biobank healthy reference cohort (ΔSLR = −0.06; 95% CI −0.08 to −0.03; *P* < 0.001). Only HCM retained a clinically meaningful excess in septal asymmetry after adjustment. Results are shown in *Figure 5*.

**Figure 5:**
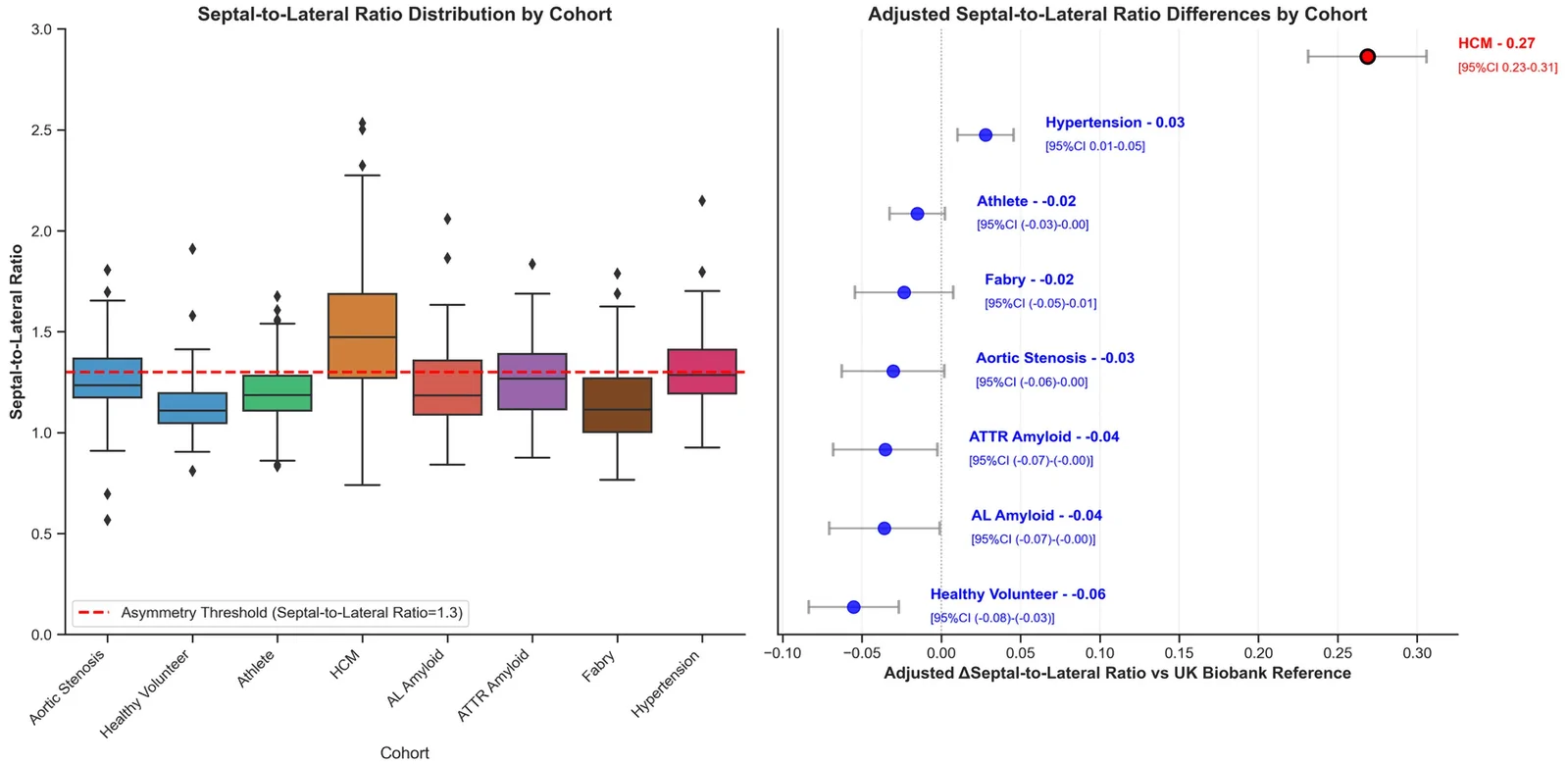
Left panel shows the distribution of the septal-to-lateral wall thickness ratio (SLR) by cohort. Boxes show median and interquartile range. The red dashed line marks the asymmetry threshold (SLR = 1.3). Right panel shows the adjusted differences in SLR (ΔSLR) vs. UK Biobank healthy reference from a multivariable linear model adjusting for age, body surface area, and sex. Dots indicate ΔSLR and bars 95% CIs. Only hypertrophic cardiomyopathy (HCM) demonstrated a materially higher SLR after adjustment (ΔSLR = +0.27; 95% CI 0.23–0.31). AL, light-chain amyloidosis; AS, aortic stenosis; ASH, asymmetric septal hypertrophy; ATTR, transthyretin amyloidosis; BSA, body surface area; HCM, hypertrophic cardiomyopathy; SLR, septal-to-lateral ratio.

### Exploratory regional morphology and discrimination analysis

Exploratory analyses of segmental wall-thickness data were performed to assess whether additional regional features differed between cohorts. The location of MWT varied significantly across groups (χ^2^ = 217.1, *P* < 0.001). Basal septal MWT was common across multiple cohorts, including HCM, hypertension, athletes, AS, and healthy volunteers, indicating that basal septal predominance was not specific to HCM. HCM showed high SLR at both basal and mid-cavity levels, whereas ATTR and AL amyloidosis showed relatively greater mid-cavity SLR than basal SLR. In discrimination analyses, adjusted SLR residual showed the strongest or near-strongest discrimination for HCM vs. amyloidosis and AS, while MWT was most discriminative for HCM vs. hypertension. These exploratory findings are presented in Supplementary data online, *Tables S2* and *S5*.

## Discussion

In this multi-cohort CMR study, we show that threshold-defined ASH is not unique to HCM. On unadjusted analysis, ASH was observed in 61% of HCM subjects but also seen across a range of hypertrophic and infiltrative phenotypes, including ATTR amyloidosis (37%), AS (26%), AL amyloidosis (23%), and Fabry disease (12%). Similar to our findings, ASH has previously been reported in ATTR amyloid,^4^ AS,^20^ and septal asymmetry without significant hypertrophy has been described in hypertension.^21^ This can blur morphological distinction between HCM and its phenocopies when ASH is considered in isolation.

However, age, sex, and body size were also independently associated with higher septal–lateral ratio in a large healthy reference population. After accounting for these demographic factors, only HCM retained a materially higher adjusted SLR (ΔSLR = +0.27), indicating that pronounced septal asymmetry is disproportionately associated with HCM. In contrast to the increased crude prevalence of ASH across several disease cohorts, the adjusted analysis showed that non-HCM disease groups had had little or no clinically meaningful positive excess in SLR relative to healthy individuals.

The discrepancy between crude ASH prevalence and adjusted continuous SLR is important. As adjusted regression assesses whether SLR is greater than expected for a subject’s age, sex, and body size, older cohorts (such as ATTR amyloidosis; mean age 73) may show a higher crude prevalence of ASH without retaining an excess in adjusted SLR.

There is inconsistency in the literature regarding the optimal SLR for defining asymmetry, with reported thresholds ranging from 1.3 to 1.5.^7,15^ The SLR threshold of 1.3 derives from echocardiography studies and it is widely used in clinical practice. However, some authors have suggested a higher ratio of 1.5 as more specific for the diagnosis of HCM.^22^ Supplementary analysis repeated using ≥1.5 is shown in Supplementary data online, *Figure S2*. Notably, the mean SLR in the healthy UK Biobank reference cohort approximated the 1.3 threshold, reinforcing that this cut-off should be interpreted as pragmatic rather than biologically absolute, particularly in older populations. Consequently, while SLR ≥1.3 remains a practical threshold for describing asymmetric morphology, categorical ASH prevalence is inherently threshold-dependent and should not be interpreted as a binary diagnostic marker. SLR is better considered as a continuous trait that should be interpreted alongside absolute wall thickness, age, sex, body size, loading conditions, tissue characterization, and broader clinical context.

An increase in the SLR with age has been recognized for >40 years^23^ and a basal septal bulge is a recognized phenomenon in older patients, affecting 18% in those >85 years.^24^ Consistent with this, we found age to be the strongest demographic correlate of an increased SLR in health. With ageing, the aorta unfolds and cardiac orientation shifts, predisposing to a basal (‘sigmoid’) septal bulge without true diffuse hypertrophy.^25^ An elevated SLR in older adults should be interpreted in light of age and body size to avoid overinterpreting ASH.

Although the mechanistic basis for septal predisposition to hypertrophy is not completely understood, its clinical relevance is clearer because septal thickening contributes to LVOTO.^26^ Mechanically, the basal septum’s proximity to the LVOT facilitates a pathological feedback loop as the initial hypertrophy causes dynamic obstruction that concentrates afterload at the septum and further stimulates localized growth.^27^ In contrast to pressure-overload or storage-related diseases which typically have more symmetric hypertrophy, athletes’ heart and hypertension manifest as mostly asymmetric remodelling without LVH, consistent with load-driven regional wall-stress effects.^28^ However, the more extreme and disproportionate septal geometry seen in HCM could suggest a disease-intrinsic morphogenetic signal rather than haemodynamic loading alone, although SLR itself should be interpreted as a morphological marker rather than direct evidence of mechanism.

In hypertension, asymmetric remodelling without LVH was common using a threshold of SLR ≥1.3 (42%), but much less frequent with a stricter threshold of SLR ≥1.5 (10%), in keeping with values previously reported.^21^ This suggests that hypertensive remodelling often produces only mild septal predominance. Consistent with this, after adjustment for BSA, sex and age, hypertension was associated with only a small increase in SLR relative to healthy volunteers (ΔSLR=+0.03; 95% CI +0.01 to +0.05). Clinically, borderline asymmetry in hypertension should therefore be interpreted cautiously and not regarded as diagnostic of HCM in isolation.

We employed machine learning analysis of CMR to enable us to scale analysis to segment thousands of studies fully automatically in a short time. The automated approach used here has previously been shown to improve precision and reduce inter- and intra-observer variability in CMR wall thickness measurement.^17^ Although the present study is based on CMR, the broader principle that septal asymmetry should be interpreted in the context of demographic factors may be relevant across imaging modalities.

Finally, although SLR is a simple and clinically usable biomarker of septal predominance, it does not capture the full diagnostic complexity of HCM or its phenocopies, including apical variants, focal hypertrophy, tissue characterization, LVOT obstruction, family history, genotype, and other regional differences outside the septal-to-lateral axis.

Exploratory segmental analyses suggested that regional hypertrophy patterns vary across disease groups. Basal septal MWT was common across several cohorts and was therefore not specific to HCM, while basal and mid-cavity SLR patterns differed between HCM and amyloidosis. These findings support the concept that richer regional or 3D shape descriptors may improve phenotyping beyond MWT and SLR. However, such modelling was beyond the scope of the present study, which focused on clinically familiar wall-thickness measures and their demographic determinants. Future research should explore whether richer quantitative descriptions of ventricular shape can improve phenotyping and diagnosis.

## Limitations

LVH was defined using maximal wall thickness, consistent with conventional HCM diagnostic frameworks. However, wall thickness is influenced not only by myocardial mass but also by chamber geometry and therefore may not fully distinguish true hypertrophy from remodelling. This is relevant when considering demographic factors such as age, sex, and body size.

This study has several limitations. First, the analysis was retrospective and assembled from previously recruited research cohorts across multiple centres. The study populations are therefore potentially subject to selection and referral bias, and the spectrum of disease severity may not have been comparable across cohorts. Some diseases may have been enriched for more advanced or phenotypically overt disease, influencing the observed prevalence of ASH and limiting generalizability.

Demographic adjustments of septal asymmetry were derived from a healthy UK Biobank reference cohort and then applied to disease cohorts. Although this allows us to interpret asymmetry to an expected healthy baseline, it assumes that the relationships between SLR and age, sex, and body size are transferable across disease states.

The difference in SLR between the UK Biobank healthy reference cohort and the local healthy volunteer cohort warrants consideration. The local healthy volunteers were younger and more selected, whereas UK Biobank provided a larger population-derived reference cohort. Given the positive association between age and SLR in UK Biobank, age differences are likely to explain part of the higher SLR observed in the UK Biobank healthy cohort. However, the lower SLR in local healthy volunteers persisted after adjustment for age, sex, and BSA, suggesting that additional factors may have contributed. These may include cohort selection, recruitment strategy and scanner or protocol differences.

LVH was defined using MWT, consistent with conventional HCM diagnostic practice, and asymmetry was defined using a pragmatic SLR of 1.3. However, several alternative definitions of hypertrophy and asymmetry are possible, including indexed LV mass, sex-specific or body size-adjusted wall-thickness thresholds, higher SLR cut-offs, or more continuous measures of ventricular geometry. Different classification frameworks may have altered the prevalence of categorical phenotypes, although the principal finding that marked septal asymmetry is disproportionately associated with HCM is unlikely to depend on any single threshold alone.

Several methodological limitations of automated wall-thickness measurement should also be acknowledged. Although the algorithm has previously been validated for CMR wall-thickness quantification and enables highly reproducible large-scale analysis, disease-specific validation was not performed equally across all comparator cohorts, including amyloidosis, AS, Fabry disease, athletes, and hypertension. Small differences in segmentation and basal slice appearances may influence septal or lateral maximum wall-thickness measurements, particularly when SLR lies close to the threshold of 1.3. For this reason, the principal inference of the study was based on adjusted continuous SLR rather than binary classification alone.

Wall thickness reflects not only myocardial mass but also ventricular geometry and cavity size and therefore may not fully distinguish true hypertrophy from remodelling.

## Conclusion

Septal asymmetry is significantly influenced by age, sex, and body size. Although threshold-defined ASH is prevalent in several diseases associated with LVH, adjusted continuous SLR showed clinically meaningful positive excess only in HCM. Importantly, these findings should be interpreted as demonstrating the diagnostic contribution of septal asymmetry rather than establishing ASH as a standalone diagnostic marker of HCM. In clinical practice, asymmetric septal morphology is interpreted alongside age, sex, body size, loading conditions, family history, ECG findings, absolute wall thickness, LVOT obstruction, LGE pattern, native T1/ECV, genotype, and extracardiac disease features. The observation that adjusted SLR is disproportionately increased in HCM therefore supports its role as one component of multiparametric assessment, rather than as an isolated criterion for distinguishing HCM from phenocopies.

## Supplementary Material

jeag203_Supplementary_Data

## Data Availability

The data underlying this article are not publicly available due to ethical and data-governance restrictions relating to participant confidentiality. Requests for access to de-identified data will be considered by the corresponding author on reasonable request and subject to the relevant approvals and data-sharing agreements.
