# Supplementary material for: Prevalence and determinants of asymmetric septal hypertrophy (ASH) in diseases of left ventricular hypertrophy (LVH)
Source: Eur Heart J Cardiovasc Imaging. 2026 Aug 3;27(10):1860–9. doi: 10.1093/ehjci/jeag203 (PMC13623422; doi:10.1093/ehjci/jeag203)
Supplement: Supplementary file 1 [file jeag203_supplementary_data.docx]

**Supplement**

**Supplement Figure 1** – Histogram plots visualize the distribution of Septal-Lateral ratio (x-axis) across the population for healthy volunteers and 7 patient groups. The black dashed line represents the cut-off values of 1.3 for Septal-Lateral ratio as asymmetry. (AL - Amyloid light chain. ATTR - Amyloid transthyretin. HCM – Hypertrophic Cardiomyopathy)

**Supplement Figure 2** - **Distribution of hypertrophy patterns using a stricter asymmetry threshold of SLR ≥1.5.** LVH was defined as maximum wall thickness ≥15 mm, and asymmetry was defined as septal-to-lateral ratio ≥1.5. Participants were grouped as normal morphology, asymmetric remodelling, symmetric hypertrophy, or asymmetric septal hypertrophy. Percentages are shown for each cohort. (AL - Amyloid light chain. ATTR - Amyloid transthyretin. HCM – Hypertrophic Cardiomyopathy).

**Supplementary Table 1 - Recruitment sources and cohort definitions.** Summary of recruitment sources for each cohort included in the study.

| **Cohort** | **Recruitment source** | **Cohort definition** |
| --- | --- | --- |
| **UK Biobank healthy reference** | UK Biobank imaging substudy | Participants taking no medications and free from hypertension, diabetes, hypercholesterolaemia, peripheral vascular disease, coronary artery disease, and cerebrovascular disease. |
| **Local healthy volunteers** | St Bartholomew’s Hospital, Royal Free Hospital, and London Heart Hospital | No history of cardiovascular disease, no regular medications, and a normal ECG. |
| **Athletes** | St Bartholomew’s Hospital, Royal Free Hospital, London Heart Hospital, and St George’s Hospital | Age >40 years; running ≥10 miles per week or cycling ≥30 miles per week for ≥10 years; participation in ≥10 endurance events over a 10-year period; no known cardiomyopathy or cardiovascular disease. |
| **Hypertension** | UK Biobank imaging substudy | Participants undergoing CMR while taking ≥3 antihypertensive medications and free from diabetes, hypercholesterolaemia, peripheral vascular disease, coronary artery disease, and cerebrovascular disease. |
| **Aortic stenosis** | St Bartholomew’s Hospital, Royal Free Hospital, and London Heart Hospital | Severe aortic stenosis, defined as peak aortic valve velocity ≥4 m/s, mean gradient ≥40 mmHg, or aortic valve area <1.0 cm². |
| **Fabry disease** | Lysosomal Storage Disorders Unit, Royal Free Hospital; Fabry400 study | Consecutive patients aged ≥18 years with a confirmed GLA mutation who underwent multiparametric CMR and same-day ECG. |
| **AL amyloidosis** | UK National Amyloidosis Centre | Typical CMR features together with biopsy-proven systemic AL amyloidosis on cardiac or non-cardiac biopsy. |
| **ATTR amyloidosis** | UK National Amyloidosis Centre | Clinical and CMR features with grade 2 or 3 cardiac uptake on 99mTc-DPD scintigraphy in the absence of monoclonal gammopathy; biopsy confirmation was required where monoclonal gammopathy was present. |
| **Hypertrophic cardiomyopathy (HCM)** | Inherited Cardiovascular Conditions service, St Bartholomew’s Hospital | Otherwise unexplained LV hypertrophy diagnosed through integration of clinical history, family history, ECG, cardiac imaging, loading conditions, and genetic testing where available, with alternative causes of LV hypertrophy excluded. |

**Supplementary Table 2 - Location of maximum wall thickness by cohort.**  Maximum wall thickness for each cohort, grouped as basal septal, mid septal, apical septal, lateral, or other (anterior/inferior).

| **Cohort** | **Basal septal** | **Mid septal** | **Apical septal** | **Lateral** | **Other (anterior/**  **inferior)** |
| --- | --- | --- | --- | --- | --- |
| Local Healthy Volunteers | 73/100 (73.0%) | 2/100 (2.0%) | 0/100 (0.0%) | 6/100 (6.0%) | 19/100 (19.0%) |
| Athletes | 225/274 (82.1%) | 6/274 (2.2%) | 0/274 (0.0%) | 10/274 (3.6%) | 33/274 (12.0%) |
| Hypertension | 314/376 (83.5%) | 14/376 (3.7%) | 1/376 (0.3%) | 5/376 (1.3%) | 42/376 (11.2%) |
| Aortic stenosis | 85/124 (68.5%) | 11/124 (8.9%) | 0/124 (0.0%) | 5/124 (4.0%) | 23/124 (18.5%) |
| Fabry disease | 78/148 (52.7%) | 10/148 (6.8%) | 3/148 (2.0%) | 24/148 (16.2%) | 33/148 (22.3%) |
| AL amyloidosis | 77/128 (60.2%) | 21/128 (16.4%) | 0/128 (0.0%) | 7/128 (5.5%) | 23/128 (18.0%) |
| ATTR amyloidosis | 86/149 (57.7%) | 39/149 (26.2%) | 0/149 (0.0%) | 8/149 (5.4%) | 16/149 (10.7%) |
| HCM | 217/356 (61.0%) | 60/356 (16.9%) | 4/356 (1.1%) | 13/356 (3.7%) | 62/356 (17.4%) |

**Supplementary Table 3** - Morphology category prevalence using SLR ≥ 1.3

| **Cohort** | **Total n** | **Normal morphology, n (%)** | **Asymmetric remodelling, n (%)** | **Symmetric hypertrophy, n (%)** | **Asymmetric septal hypertrophy (ASH), n (%)** |
| --- | --- | --- | --- | --- | --- |
| UK Biobank healthy reference | 4020 | 2619 (65.1%) | 1297 (32.3%) | 25 (0.6%) | 79 (2.0%) |
| Local healthy volunteers | 100 | 90 (90.0%) | 9 (9.0%) | 1 (1.0%) | 0 (0.0%) |
| Athletes | 274 | 217 (79.2%) | 50 (18.2%) | 2 (0.7%) | 5 (1.8%) |
| Hypertension | 376 | 188 (50.0%) | 158 (42.0%) | 15 (4.0%) | 15 (4.0%) |
| Aortic stenosis | 124 | 38 (30.6%) | 15 (12.1%) | 39 (31.5%) | 32 (25.8%) |
| Fabry disease | 148 | 93 (62.8%) | 9 (6.1%) | 28 (18.9%) | 18 (12.2%) |
| AL amyloidosis | 128 | 23 (18.0%) | 12 (9.4%) | 63 (49.2%) | 30 (23.4%) |
| ATTR amyloidosis | 149 | 10 (6.7%) | 13 (8.7%) | 71 (47.7%) | 55 (36.9%) |
| Hypertrophic cardiomyopathy | 356 | 22 (6.2%) | 31 (8.7%) | 87 (24.4%) | 216 (60.7%) |

**Supplementary Table 4** - Morphology category prevalence using SLR ≥ 1.5

| **Cohort** | **Total n** | **Normal morphology, n (%)** | **Asymmetric remodelling, n (%)** | **Symmetric hypertrophy, n (%)** | **Asymmetric septal hypertrophy (ASH), n (%)** |
| --- | --- | --- | --- | --- | --- |
| UK Biobank healthy reference | 4020 | 3709 (92.3%) | 207 (5.1%) | 77 (1.9%) | 27 (0.7%) |
| Local healthy volunteers | 100 | 97 (97.0%) | 2 (2.0%) | 1 (1.0%) | 0 (0.0%) |
| Athletes | 274 | 259 (94.5%) | 8 (2.9%) | 5 (1.8%) | 2 (0.7%) |
| Hypertension | 376 | 316 (84.0%) | 30 (8.0%) | 24 (6.4%) | 6 (1.6%) |
| Aortic stenosis | 124 | 49 (39.5%) | 4 (3.2%) | 65 (52.4%) | 6 (4.8%) |
| Fabry disease | 148 | 100 (67.6%) | 2 (1.4%) | 42 (28.4%) | 4 (2.7%) |
| AL amyloidosis | 128 | 33 (25.8%) | 2 (1.6%) | 84 (65.6%) | 9 (7.0%) |
| ATTR amyloidosis | 149 | 20 (13.4%) | 3 (2.0%) | 115 (77.2%) | 11 (7.4%) |
| Hypertrophic cardiomyopathy | 356 | 34 (9.6%) | 19 (5.3%) | 158 (44.4%) | 145 (40.7%) |

**Supplementary Table 5. Regional septal-to-lateral ratio and discrimination of HCM from comparator cohorts**

Basal, mid-cavity, and overall septal-to-lateral wall-thickness ratio (SLR) by cohort as mean ± standard deviation. Panel B reports area under the receiver-operating-characteristic curve (AUC) values with 95% confidence intervals for discrimination of hypertrophic cardiomyopathy (HCM) from comparator cohorts. AL and ATTR amyloidosis are analysed separately.

**Panel A. Regional SLR by cohort**

| **Cohort** | **n** | **Basal SLR** | **Mid-cavity SLR** | **Overall SLR** |
| --- | --- | --- | --- | --- |
| **UK Biobank healthy reference** | 4,020 | 1.25 ± 0.16 | 1.24 ± 0.16 | 1.25 ± 0.16 |
| **Local healthy volunteers** | 100 | 1.13 ± 0.15 | 1.18 ± 0.14 | 1.13 ± 0.15 |
| **Athletes** | 274 | 1.20 ± 0.14 | 1.16 ± 0.10 | 1.20 ± 0.14 |
| **Hypertension** | 376 | 1.30 ± 0.17 | 1.32 ± 0.18 | 1.30 ± 0.16 |
| **Aortic stenosis** | 124 | 1.26 ± 0.16 | 1.25 ± 0.22 | 1.26 ± 0.18 |
| **Fabry disease** | 148 | 1.16 ± 0.20 | 1.20 ± 0.17 | 1.15 ± 0.19 |
| **AL amyloidosis** | 128 | 1.23 ± 0.22 | 1.44 ± 0.22 | 1.22 ± 0.20 |
| **ATTR amyloidosis** | 149 | 1.25 ± 0.19 | 1.50 ± 0.29 | 1.26 ± 0.19 |
| **HCM** | 356 | 1.53 ± 0.33 | 1.48 ± 0.38 | 1.50 ± 0.34 |

**Panel B. Discrimination of HCM from comparator cohorts**

| **Comparison** | **Variable** | **n (HCM)** | **n (comparator)** | **AUC** | **95% CI** |
| --- | --- | --- | --- | --- | --- |
| **HCM vs All non-HCM disease** | **MWT** | 356 | 925 | 0.776 | 0.751–0.801 |
|  | **SLR** | 356 | 925 | 0.738 | 0.705–0.772 |
|  | **Septal MWT** | 356 | 925 | 0.780 | 0.755–0.804 |
|  | **Lateral MWT** | 356 | 925 | 0.618 | 0.586–0.651 |
|  | **Adjusted SLR residual** | 356 | 925 | 0.780 | 0.748–0.811 |
|  | **Basal SLR** | 356 | 925 | 0.778 | 0.747–0.808 |
|  | **Mid-cavity SLR** | 356 | 925 | 0.619 | 0.581–0.656 |
| **HCM vs AL amyloidosis** | **MWT** | 356 | 128 | 0.641 | 0.585–0.697 |
|  | **SLR** | 356 | 128 | 0.767 | 0.723–0.811 |
|  | **Septal MWT** | 356 | 128 | 0.647 | 0.590–0.704 |
|  | **Lateral MWT** | 356 | 128 | 0.409 | 0.353–0.465 |
|  | **Adjusted SLR residual** | 356 | 128 | 0.800 | 0.758–0.841 |
|  | **Basal SLR** | 356 | 128 | 0.803 | 0.761–0.845 |
|  | **Mid-cavity SLR** | 356 | 128 | 0.512 | 0.460–0.565 |
| **HCM vs ATTR amyloidosis** | **MWT** | 356 | 149 | 0.429 | 0.371–0.486 |
|  | **SLR** | 356 | 149 | 0.730 | 0.686–0.774 |
|  | **Septal MWT** | 356 | 149 | 0.429 | 0.371–0.487 |
|  | **Lateral MWT** | 356 | 149 | 0.311 | 0.258–0.364 |
|  | **Adjusted SLR residual** | 356 | 149 | 0.798 | 0.759–0.837 |
|  | **Basal SLR** | 356 | 149 | 0.773 | 0.732–0.814 |
|  | **Mid-cavity SLR** | 356 | 149 | 0.460 | 0.407–0.513 |
| **HCM vs Aortic stenosis** | **MWT** | 356 | 124 | 0.711 | 0.656–0.766 |
|  | **SLR** | 356 | 124 | 0.731 | 0.686–0.776 |
|  | **Septal MWT** | 356 | 124 | 0.731 | 0.680–0.783 |
|  | **Lateral MWT** | 356 | 124 | 0.502 | 0.447–0.556 |
|  | **Adjusted SLR residual** | 356 | 124 | 0.804 | 0.765–0.844 |
|  | **Basal SLR** | 356 | 124 | 0.780 | 0.739–0.822 |
|  | **Mid-cavity SLR** | 356 | 124 | 0.694 | 0.646–0.742 |
| **HCM vs Hypertension** | **MWT** | 356 | 376 | 0.961 | 0.949–0.973 |
|  | **SLR** | 356 | 376 | 0.697 | 0.658–0.737 |
|  | **Septal MWT** | 356 | 376 | 0.959 | 0.946–0.972 |
|  | **Lateral MWT** | 356 | 376 | 0.836 | 0.807–0.865 |
|  | **Adjusted SLR residual** | 356 | 376 | 0.756 | 0.720–0.792 |
|  | **Basal SLR** | 356 | 376 | 0.739 | 0.703–0.776 |
|  | **Mid-cavity SLR** | 356 | 376 | 0.639 | 0.597–0.680 |
| **HCM vs Fabry disease** | **MWT** | 356 | 148 | 0.827 | 0.779–0.875 |
|  | **SLR** | 356 | 148 | 0.830 | 0.794–0.867 |
|  | **Septal MWT** | 356 | 148 | 0.833 | 0.786–0.879 |
|  | **Lateral MWT** | 356 | 148 | 0.653 | 0.600–0.707 |
|  | **Adjusted SLR residual** | 356 | 148 | 0.784 | 0.744–0.824 |
|  | **Basal SLR** | 356 | 148 | 0.855 | 0.820–0.890 |
|  | **Mid-cavity SLR** | 356 | 148 | 0.756 | 0.714–0.798 |
